# Identification and validation of novel and more effective choline kinase inhibitors against *Streptococcus pneumoniae*

**DOI:** 10.1038/s41598-020-72165-6

**Published:** 2020-09-22

**Authors:** Tahl Zimmerman, Valerie Chasten, Juan Carlos Lacal, Salam A. Ibrahim

**Affiliations:** 1 Food Microbiology and Biotechnology Laboratory, Department of Family and Consumer Sciences, College of Agriculture and Environmental Sciences, North Carolina A&T State University, 1601 East Market Street, Greensboro, NC 27411 USA; 2 Instituto de Investigaciones Biomédicas, Consejo Superior de Investigaciones Científicas, c/ Arturo Duperier 4, 28029 Fuenlabrada, Madrid Spain

**Keywords:** Drug discovery, Target validation

## Abstract

*Streptococcus pneumoniae* choline kinase (sChoK) has previously been proposed as a drug target, yet the effectiveness of the first and only known inhibitor of sChoK, HC-3, is in the millimolar range. The aim of this study was thus to further validate sChoK as a potential therapeutic target by discovering more powerful sChoK inhibitors. LDH/PK and colorimetric enzymatic assays revealed two promising sChoK inhibitor leads RSM-932A and MN58b that were discovered with IC50 of 0.5 and 150 μM, respectively, and were shown to be 2–4 magnitudes more potent than the previously discovered inhibitor HC-3. Culture assays showed that the minimum inhibitory concentration (MIC) of RSM-932A and MN58b for *S. pneumoniae* was 0.4 μM and 10 μM, respectively, and the minimum lethal concentration (MLC) was 1.6 μM and 20 μM, respectively. Western blot monitoring of teichoic acid production revealed differential patterns in response to each inhibitor. In addition, both inhibitors possessed a bacteriostatic mechanism of action, and neither interfered with the autolytic effects of vancomycin. Cells treated with MN58b but not RSM-932A were more sensitive to a phosphate induced autolysis with respect to the untreated cells. SEM studies revealed that MN58b distorted the cell wall, a result consistent with the apparent teichoic acid changes. Two novel and more highly potent putative inhibitors of sChoK, MN58b and RSM-932A, were characterized in this study. However, the effects of sChoK inhibitors can vary at the cellular level. sChoK inhibition is a promising avenue to follow in the development of therapeutics for treatment of *S. pneumoniae*.

## Introduction

The effectiveness of current therapeutics for pathogens such *as S. pneumoniae* has dropped with the emergence of resistant strains. Consequently, new methods for treating *S. pneumoniae* have to be developed continuously. This includes establishing novel targets for drug discovery efforts. Recently, choline kinase (ChoK) has been proposed as a drug target for Gram positive species generally^1^ after the first ChoK inhibitor capable of inhibiting the growth of *S. pneumoniae* was discovered^2,3^.

Like its eukaryotic counterparts, bacterial ChoKs phosphorylate choline (Cho) into phosphocholine (PCho). PCho is a precursor molecule that is utilized in the production of two types of teichoic acids: lipoteichoic acid (LTA) and cell wall teichoic acid (CTA)^4,5^. In *S. pneumoniae,* both LTA and CTA consist of the same types of polysaccharides; however, LTA is attached to a lipid and embedded in the cell membrane, and CTA is attached to the cell wall peptidoglycan layer in the cell wall^6^. LTA is an important virulence factor^7^, and production of this molecule has been validated as a drug target^8^.

Choline is an essential nutrient for *S. pneumoniae*^9^, and the choline kinase of *S. pneumoniae* (sChoK) is an essential enzyme^10^. The sChoK enzyme is an element of the pathway that mediates the decoration of teichoic acids with PCho via the intermediary CDP-choline. Genes that are part of this pathway are expressed via the *lic* gene locus. The LicB gene expresses a Cho transporter which collects Cho from the external environment. LicA codes for sChoK, which phosphorylates Cho into PCho. LicC is a gene coding for cytidylyl transferase, which converts PCho into CDP-choline. The LicD1 and LicD2 PCho transferases remove PCho from CDP-choline and attach them to *N*-acetylgalactosamine (GalNAc) residues located on pre-teichoic acid glycan subunits^11^. These residues are then polymerized by an unidentified protein to form teichoic acid which is^11^ transported across the cell membrane acid flippase TacF. The TacL ligase attaches teichoic polymers to a glycolipid anchor to form LTA^7^. Meanwhile, teichoic acid polymers are cross-linked to peptidoglycan to form WTA by the action of LCP phosphotransferases^11^.

**Figure 1:**
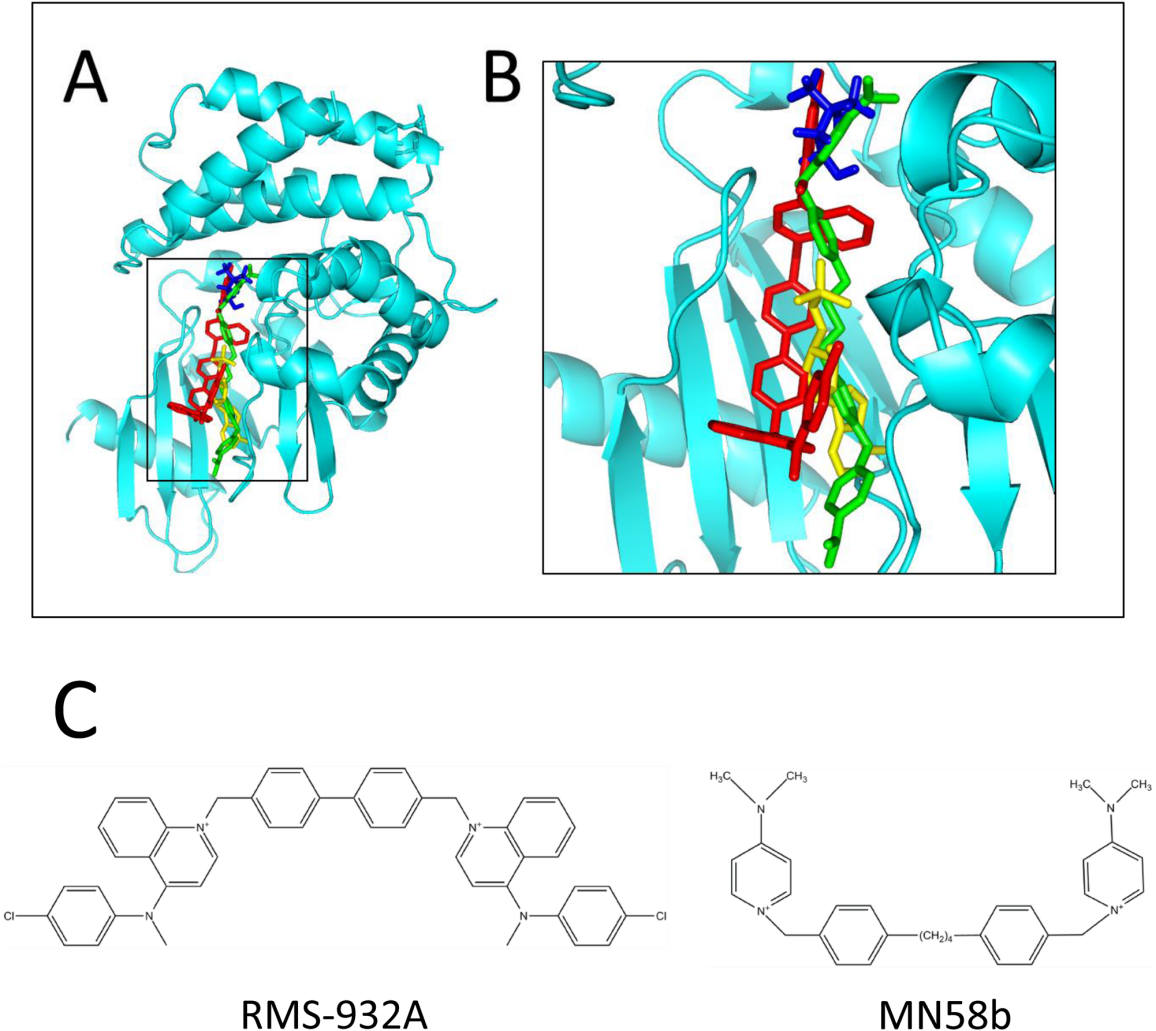
(**A**) MN58b (green) and RSM-932A (red) docked onto the crystal structure of apo-sChoK (accession #4R77) as well as the positions of the natural substrates choline (blue) and AMP (yellow); (**B**) a close up view of the binding sites; (**C**) Structures of RSM-932A (1,1′-([1,1′-biphenyl]-4,4′-diylbis(methylene))bis(4-((4-chlorophenyl)(methyl)amino)quinolin-1-ium) bromide) and MN58b (1,4-[4–4′-Bis-{[4-(dimethylamine)pyridinium-1-yl]methyl}diphenyl]butane dibromide).

The enzyme of *S. pneumoniae* has previously been proposed as a drug target^3^. However, the sChoK inhibitor originally tested, Hemicholinium-3 (HC-3), was weak: HC-3 had IC_50_^12^ and minimum inhibitory concentration (MIC) values in the millimolar range^3^. The aim of this study was to discover stronger inhibitors of sChoK in order to further establish sChoK as a target. Here, we present two compounds which strongly inhibit sChoK activity and *S. pneumoniae* growth and viability: MN58b and RSM-932A (Fig. 1C)^13,14^, two compounds known to inhibit human choline kinase (hChoK)^14^.

Using *S. pneumoniae* as a model, we present evidence to demonstrate that employing sChoK inhibitors is a promising strategy for blocking the growth of *S. pneumoniae* cells. These inhibitors, in turn, were found to have downstream effects on teichoic acid production and assembly, on cell wall shape, and on the sensitivity of the cell to autolysis.

## Methods and materials

### Recombinant sChoK expression and purification

A pET28a/sChoK construct was generously provided to us by the group of Dr. Yuxing Chen^15^ and transformed into BL21 (DE3) cells. Cells were cultured at 200 rpm at 37 °C until an O.D._600_ of 0.6 was reached and then induced with 1 mM IPTG overnight at 25 °C. Cells were then harvested by centrifugation and stored at − 20 °C. Cell lysis was performed by suspending each gram of pellet with 5 mL of B-PER Complete Bacterial Protein Extraction Reagent (Thermo-Scientific) per gram of cell. The enzyme sChoK was purified using HisPur Ni-NTA Resin (Thermo Scientific).

### Determining steading state kinetic constants by LDH/PK

Purified sChoK was used in these assays. Steady-state enzymatic assays were monitored with the pyruvate kinase/lactate dehydrogenase (PK/LDH) reaction. The steady-state rate of ADP formation was calculated using an extinction coefficient of 6,200 M^−1^ cm^−1^ to determine linear reductions in NADH concentrations that were observed by absorbance at 340 nm. Measurements were carried out at 25 °C in a UV-Star 96-well plate (Greiner) placed in a Synergy HT microplate reader (Biotek). Each well contained 200 µL of a solution containing 22 nM sChoK enzyme, 100 mM Tris pH 8, 10 mM MgCl_2_, 150 mM NaCl, 150 mM KCl, 100 mM NADH, 0.66 mM phosphoenolpyruvate, 20 units of LDH, and 10 units of PK, choline. ATP. K_m_ and V_max_ values were derived by plotting initial velocities against the substrate concentration and fitting this data into Eq. (1).

1 $$ {\text{v}}_{0} = ({\text{V}}_{\max } [{\text{S}}])/({\text{K}}_{{\text{m}}} + [{\text{S}}]) $$

where v_0_ is the initial velocity [S] = substrate concentration; V_max_ = maximum velocity; and K_m_ is the concentration at half-maximal velocity. The k_cat_ was calculated by dividing the V_max_ by the enzyme concentration.

### Determining IC_50_ by LDH/PK

Using the conditions described above, the concentration of choline was set to the K_m_ and increasing amounts of inhibitor were tested. Percent inhibition was determined. The IC50 was determined as the concentration of inhibitor required to reach 50% inhibition.

### Determining IC_50_ colorimetrically

When using cell extracts such as BL21 (DE3) extracts containing sChoK or *S. pneumoniae* extracts as the enzyme source, a colorimetric method was used to quantify Cho consumption and PCho production as described previously^16^.

### Determining MN58b and RSM-932A competitivity by LDH/pK

The mechanism of action of the inhibitors with respect to each substrate was determined using a previously described method^13,14^. Briefly, using the IC_50_ of each inhibitor determined at the K_m_ of choline, the concentration of one substrate was kept constant, and initial velocities were measured while varying the second substrate in the presence and absence of the inhibitor. The percentage of inhibition (%I) was determined by dividing each initial velocity in the presence of the inhibitor with its corresponding initial velocity in the absence of compound. %I was plotted against concentration and fitted using a two-parameter-fit nonlinear regression algorithm of GnuPlot v 4.3 into Eq. (2).

2 $$ \% {\text{I}} = 100 \, \times (([{\text{I}}]/{\text{Ki}} + [{\text{S}}][{\text{I}}]/\alpha {\text{KsKi}}/(1 + [{\text{S}}]/{\text{Ks}} + [{\text{I}}]/{\text{Ki}} + [{\text{S}}][{\text{I}}]/\alpha {\text{KsKi}})) $$

where [S] is the concentration of the varied substrate, [I] is the concentration of inhibitor, Ks is the dissociation constant of the substrate (assumed to be equal to the K_m_), Ki is the dissociation constant of the inhibitor, and α is a numerical constant that measures the effect the substrate has on the binding of the inhibitor and vice versa.

### Docking of MN58b and RSM-932A onto sChoK

With regard to the kinetic data, MN58b and RSM-932A were docked into the active site and choline site alone, respectively using apo-sChoK structure as a model (RCSB accession #4R77). The entire active site was defined as all of the residues of sChoK known from the crystal structure of sChoK to make contact with both ATP (43, 90, 183, 89, 91, 31, 818, and 194) and choline (residue numbers 197, 251, 254, 268, 178, 213, 29, 176), while the choline site was defined as only those residues making contact with choline.

The molecular structure of MN58b was defined using the JSME molecular editor (https://cactus.nci.nih.gov/translate/editor.html), and a .pdb file for this molecule was generated using the PRODRG server (https://davapc1.bioch.dundee.ac.uk/cgi-bin/prodrg/submit.html). The resulting structure was then inspected using Pymol. The RSM-932A .SDF structure file was downloaded from the RCSB Protein Data bank (https://www.rcsb.org, accession number 5W60) and converted to a .pdb using the online SMILES translator (https://cactus.nci.nih.gov/translate/). Docking was performed using the Patchdock server. (https://bioinfo3d.cs.tau.ac.il/PatchDock/). The top ten structures were then refined using Firedock (https://bioinfo3d.cs.tau.ac.il/FireDock/), and the top solution from this output was selected as the most likely final structure of the complexes. Protein alignments and figures were performed using Pymol, and Fig. 1A and B were produced with this program as well.

### Determining minimum inhibitory concentration (MIC) and minimum lethal concentration (MLC)

Seven μL of a glycerol stock of the *S. pneumoniae* R6 strain was used to inoculate 7 mL of Brain Heart Infusion broth (BHI, Accumedia, Sydney, Australia) supplemented with 5 units/mL catalase (BHI-CAT). This starter culture was incubated at 37 °C in a water bath until an optical density at 610 nm (OD_610_) was reached. Forty μL of this starter culture was used to inoculate 40 mL of BHI-CAT media with or without varying concentrations of inhibitor. This culture was then incubated at 37 °C in water bath until an OD_610_ of about 1.0 was reached in the control.

MIC was determined as the concentration inhibitor at which an O.D._610_ of 0 was measured at the end of culture time. MLC was defined as the concentration at which 99.9% of cells were killed at the end of culturing time as determined by CFU counts using Meuller–Hinton plates.

### Western blot detection of lipoteichoic acid

R6 cells were cultured to saturation in either BHI-cat alone or BHI-cat 0.5 MIC of MN58b or RSM-932A. One milliliter (1 mL) of cell samples was centrifuged at 3,000*g*, sonicated 3 × 2 min on ices. Western blots were performed as described^3^ while total protein on the membrane was measured by fluorescence according to Quickstain instructions.

### Scanning electron microscopy monitoring of *S. pneumoniae* cells treated with MN58b or RSM-932A

Scanning electron microscopy was performed as previously reported^17^.

## Results

### sChok kinetic constants

Kinetic constants were measured for sChoK: K_m_, and k_cat_ values were 130 ± 36 µM, 3.14 ± 0.24 s^−1^, respectively. These results were comparable to previously reported values^15^. This initial information was used to design additional steady state experiments, such as IC_50_ measurements and determinations of drug competitivity.

### IC_50_ results by LDH/PK and colorimetric methods

The IC_50_s of each drug against sChoK were measured using two methods: the indirect ldh/pk method that relied on recombinant sChoK and which indirectly measured consumption of ATP^14^ and a previously reported colorimetric method that relied *on BL21 *(*DE3*) extracts expressing recombinant sChoK, which directly measured the consumption of choline and the production of phosphocholine^16^. Results are shown in Table 1. Inhibitors were similarly effective in a complex cell extract and in a simplified system relying on a purified enzyme. Both MN58b and RSM-932A had IC_50s_ that fell within the µM range, which was an improvement over the IC_50_ previously reported for the sChoK inhibitor HC-3 (IC_50_ of 2.7 mM)^12^. RSM-932A was stronger that MN58b by 1–2 orders of magnitude.

**Table 1:** MICs, MLCs, and IC_50_s for RSM-932A and MN58b.

	MIC (μM)	MLC (μM)	IC50 (μM) (ldh/pK)	IC50 (μM) (colorimetric)
RSM-932A	0.4	1.6	22	0.5
MN58b	10	20	645	225

### MIC and MLC results

Both drugs had MICs and MLCs in the low micromolar ranges (see Table 1), making them much more powerful inhibitors of *S. pneumoniae* growth than the previously reported sChoK inhibitor HC-3, which had an MIC of 5,400 µM^3^. To our knowledge, other than HC-3, no other sChoK inhibitors have ever been reported, making MN58b and RSM-932A the most effective sChoK inhibitors thus far reported both on the level of enzyme inhibition and inhibition of cell growth.

### MN58b is competitive with both choline and ATP while RSM-932A is competitive with choline alone

**Figure 2:**
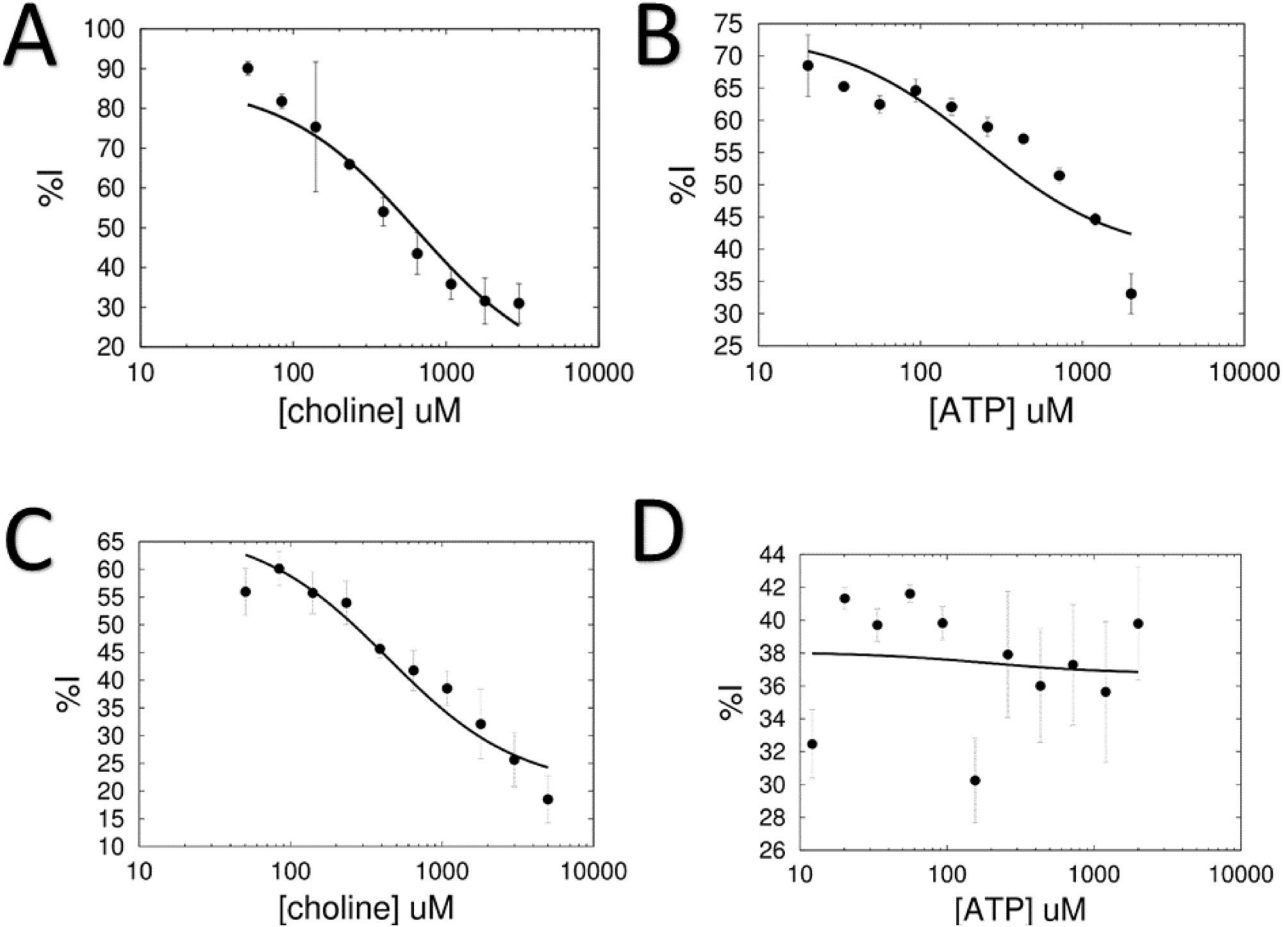
Substrate-inhibitor mechanistic analyses: (**A**) choline vs MN58b; (**B**) ATP vs MN58b; (**C**) choline vs RSM-932A; and (**D**) ATP vs RSM-932A.

A mechanistic analysis was performed for each inhibitor by performing steady-state enzymatic reactions with sChoK in which one substrate was varied while the other substrate was fixed both in assays with and without inhibitor fixed at a concentration equal to its IC_50_. sChoK inhibition was calculated as a percentage (%I) calculated by dividing the initial velocity at each substrate concentration with inhibitor by counterpart initial velocities in the absence of inhibitor. %I values were plotted against substrate concentration. The resulting data was fitting to an Eq. (2) and *K*_*i*_ and α values calculated. In the case of MN58b, %I went down as the concentration of substrates choline (Fig. 2A) or ATP (Fig. 2B) went up, indicating that inhibitor binding was antagonistic to the binding of both choline and ATP (and vice versa) In contrast, with RSM-932A, an increase in choline marked a parallel increase in %I, while an increase in ATP showed no observable trend (Fig. 2C,D, respectively), meaning that inhibitor binding was antagonistic to choline and non-competitive with ATP.

As a measure of the effect of each substrate on inhibitor binding, α and K_i_ (dissociation constant) values were derived for both inhibitors by fitting %I values according to Eq. (1) (see Table 2, “Methods and materials”). The α values calculated for MN58b were low with respect to both choline and ATP; that is, they did not approach infinity. These results supported a mixed-inhibition mechanism for these compounds (not completely competitive). Changes in the steady state constants K_m_ and V_max_ were also determined in the presence and absence of drug by fitting initial velocity and substrate values according to Eq. (1). Observed increases in K_m_ values coupled with decreases in V_max_ values supported a mixed inhibition model in which the inhibitor binding displaced both choline and ATP. Meanwhile, α values derived for RSM-932A were low in the case of choline (indicating a mixed inhibition model) but approached a value of 1 in the case of ATP, which was consistent with a noncompetitive mechanism of action. In the case of ATP, only a slight drop in K_m_ values was observed between the presence and absence of inhibitor coupled with a drop in V_max_, which supports a non-competitive model. In the case of choline, a decrease in both V_max_ and K_m_ was observed, supporting a mixed-mode of inhibition in which only choline is displaced by the inhibitor.

**Table 2:** Dissociation constants (Ki), alpha coefficient values and ratio of Michaelis–Menten kinetic constants K_m_ and V_max_ constants derived from substrate-inhibitor competition assays. (A) Inhibitor vs ATP (B) Inhibitor vs choline.

	Ki (μM)	α	Km_inh_/Km_free_	Vmax_inh_/Vmax_free_	Mechanism of Inhibition
(A)					
RSM-932A	35.8 ± 3.2	1.1 ± 0.17	0.92	0.61	Noncompetitive
MN58b	182.3 ± 28.1	6.0 ± 2.2	3.48	0.76	Mixed
(B)					
RSM-932A	10.6 ± 0.9	8.1 ± 1.8	3.08	0.76	Mixed
MN58b	123.8 ± 19.2	a = 59.2 ± 90.9	2.3	0.69	Mixed

### MN58b and RSM-932A could fit into the active site of sChoK in a differential manner

From the kinetic studies, it was concluded that MN58b and RSM-932A displaced choline, but only MN58b displaced ATP. These results do not indicate whether or not substrate displacement takes place via direct binding to the active site of the enzyme or via inhibitor binding to the allosteric site. Nevertheless, it would be interesting to determine whether or not the inhibitor binding site could be in the active site. That is, could MN58b *directly* displace both choline and ATP and could RSM-932A *directly* displace choline? Under the hypothesis that direct displacement took place, MN58b was docked onto the sChoK protein site at the ATP and choline binding sites, and RSM-932A was docked onto the choline binding site (Fig. 1A,B) The drugs were docked onto the sChok protein structure using the program Patchdock^18^. While both MN58b and RSM-932A could be seen binding to a site overlapping with the binding site of choline, only MN58b entered and entirely overlapped with an AMP molecule used to model the structure of sChoK with ATP.

### MN58b and RSM-932A do not provoke or prevent autolysis

**Figure 3:**
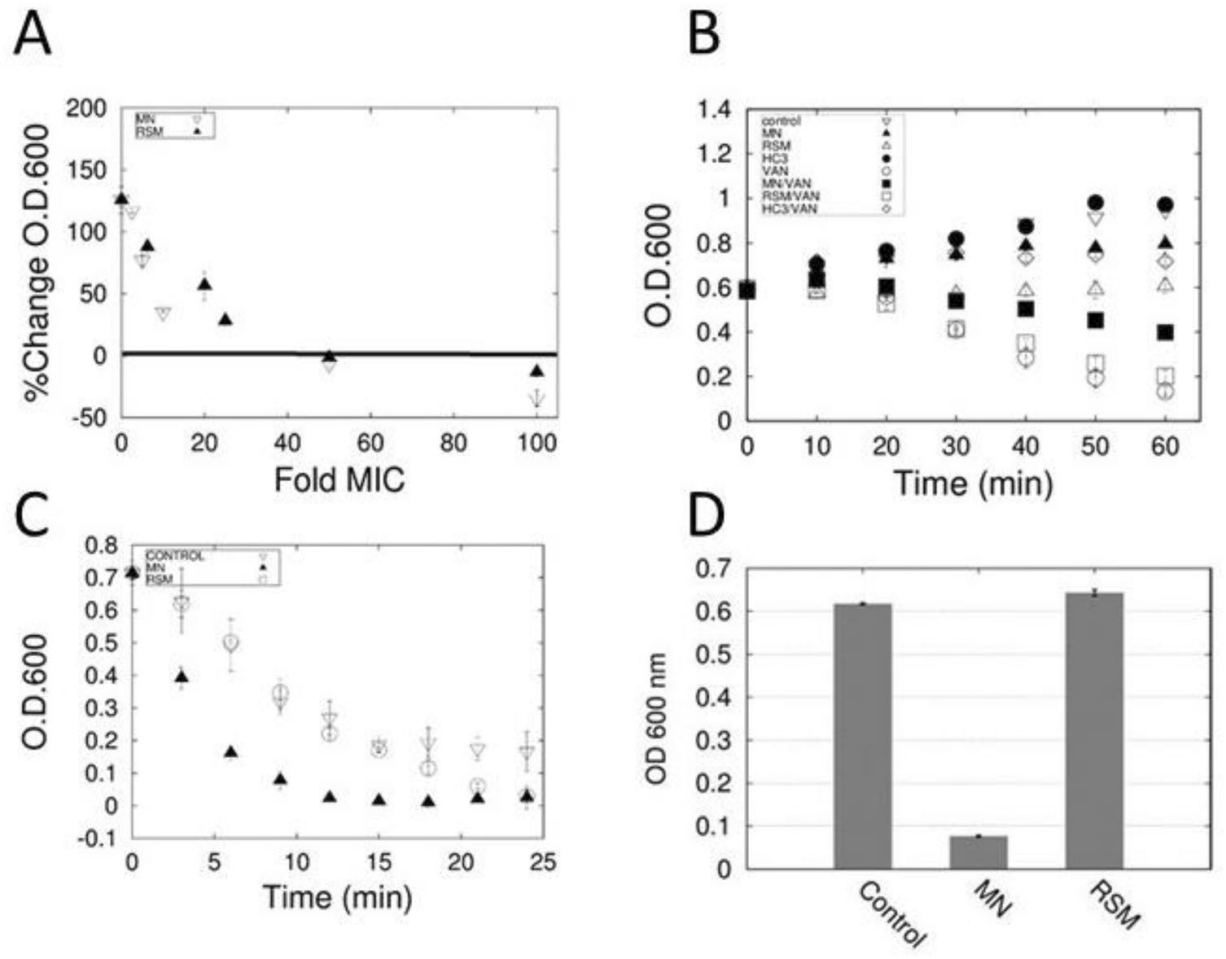
Effect of drugs on the autolytic process. (**A**) Percent changes in O.D. were measured in cultures of *S. pneumoniae* exposed to various concentrations of drugs expressed as multiples of MIC. Percent changes were calculated by comparing initial and final O.D. values. (**B**) O.D. changes in cells treated with vancomycin (VAN) in the presence and absence of HC-3 (HC3), MN58b (MN), and RSM-932A (RSM). (**C**) Detergent induced lysis of cells grown to saturation in the presence of 0.5 MIC MN58b and RSM-932A. (**D**) Phosphate induced lysis of cells grown to saturation in the presence of 0.5 MIC MN58b and RSM-932A.

By testing the effects of incubation on optical densities of cell cultures grown to an O.D. of 0.500 at different concentrations of inhibitor, we found that that MN58b and RSM-932A were bacteriostatic up to a concentration of 50 × MIC after which they become bacteriolytic (Fig. 3A).

Autolysis is a process of cell lysis and death that is mediated by LTAs via choline binding autolysins^19^ such as LytA. The choline analog and sChoK inhibitor HC-3 had previously been found to attenuate the autolytic process in *Lactobacillus reuteri* cells^17^. In light of the non-specificity observed with HC-3 (being able to interfere with both ChoKs and the autolytic process), it seemed logical to check the specificity of MN58b and RSM-932A for sChoK inhibition. The interaction of MN58b, RSM-932A, and HC-3 with bacteriolytic antibiotic vancomycin was determined (Fig. 3B). As expected, HC-3 prevented cell lysis provoked by vancomycin, but MN58b and RSM-932A had minimal to no effect, indicating that the latter drugs were more specific sChoK inhibitors than HC-3. The non-specificity of HC-3 may help to explain its weakness against sChoK.

### MN58b sensitizes *S. pneumoniae* cells to autolysis while RSM-932A does not

Given the observation that MN58b and RSM-932A were sChoK inhibitors, our expectation was that cells treated with these inhibitors would show a decrease in overall LTA content because a decrease in phosphocholine availability would reduce the overall production of LTA residues and, by extension, LTA molecules. A reduction in LTA content, in turn, should help desensitize cells to autolytic induction. Although autolysin proteins would be released, they would not be able to dock on the cell surface because the autolysin choline binding domains would have fewer choline containing LTA molecules with which to interact. Both LTA reduction and cell sensitization had previously been observed with HC-3^17^. Surprisingly, cells grown in the presence of 0.5 MIC RSM-932A were equally sensitive to autolysis as untreated controls, while MN58b actually primed the cells to be autolyzed (Fig. 3C,D). These unexpected results demonstrated that the effect of MN58b and RSM-932A on LTA production and sensitivity to autolysis were not as predictable as the results observed with HC-3.

### Western blot detection of LTA in cells treated with MN58 and RSM-932A

**Figure 4:**
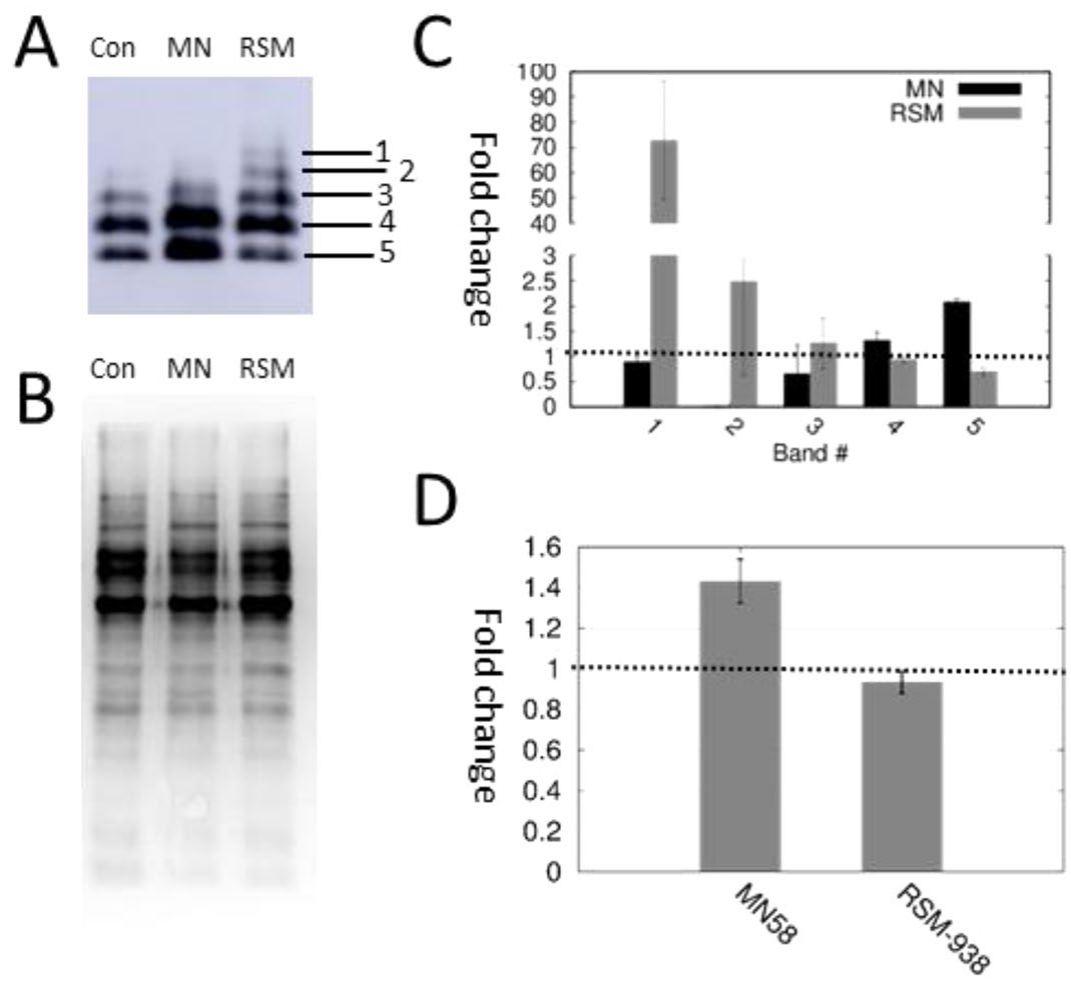
Effect of the drugs on LTA production. (**A**) Western blot of total LTA from *S. pneumoniae* cells grown in the presence or absence of MN58b (MN) or RSM-932A (RSM). The western blot is cropped to focus on LTA species. Different species of LTA are numbered 1–5, with 1 being the largest in molecular weight and 5 being the smallest (see Supplementary Material for full western). (**B**) Total protein signal from the same membrane, used as loading and membrane transfer control. (**C**) Analysis of fold changes in the different species of LTA (1–5). 1 × fold change is marked with a dotted line. (**D**) Total fold change of LTA. 1 × fold change is indicated with a dotted line.

In order to quantify LTA production, western blots were performed on extracts of cells grown to saturation in the presence of 0.5 MIC MN58b or RSM-932A. LTA band patterns clearly differed between treatments. With MN58b, a marked increase in total LTA production was observed (Fig. 4A,B,D; see Supplementary Figure). In addition, there was a marked increase in the smaller sizes of LTA (Fig. 4A–C). It is important to note that each size band in an LTA western blot differs from the lower one by a single LTA residue (Fig. 4A)^19^. This result indicated that MN58b was dysregulating both LTA production and subunit polymerization. Meanwhile, with RSM-932A, overall production remained unchanged (Fig. 4D) while larger size polymers were favored (Fig. 4C). However, this result was unexpected given our original hypothesis that sChoK inhibitors should limit production of LTAs and is even more surprising because each inhibitor has a distinct effect on LTA patterns observed.

### MN58b but not RSM-932A causes deformation of the *S. pneumoniae* cell wall

**Figure 5:**
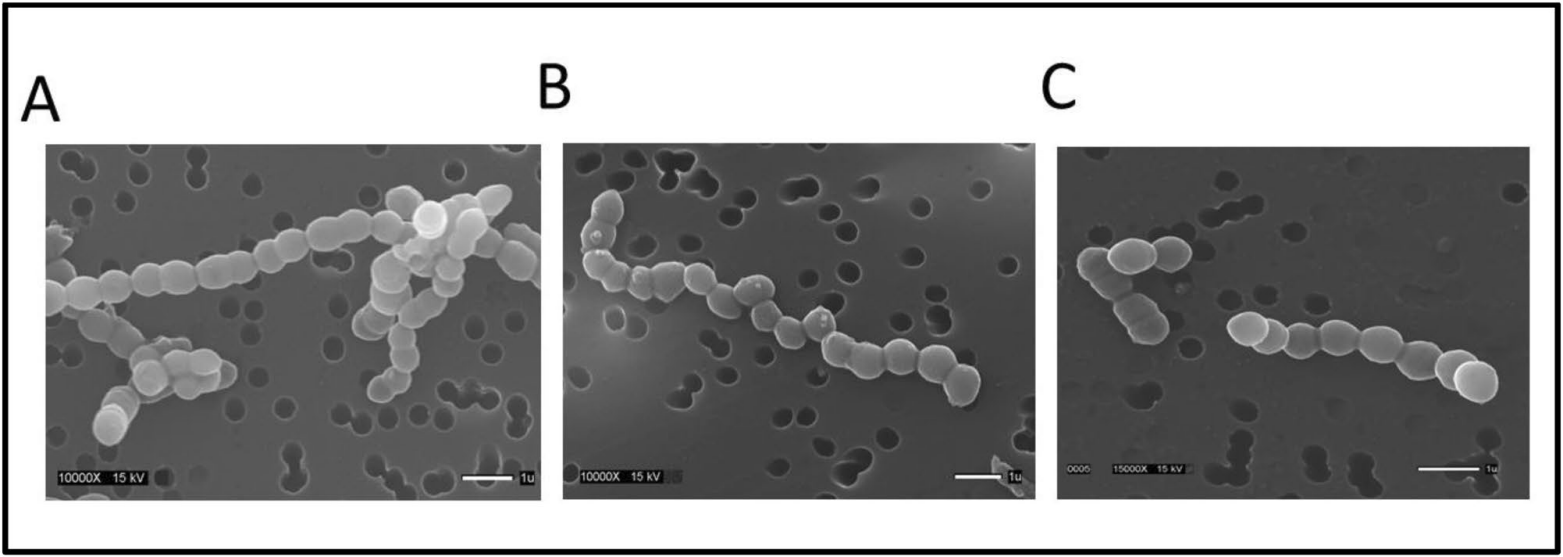
Scanning electron microscopy of R6 cells grown in the presence or absence of sChoK inhibitors at 0.5 MIC: (**A**) untreated control; (**B**) treated with MN58b; and (**C**) treated with RSM-932A.

In order to find out if these distinct effects on LTA production extended to effects on the shape of the cell wall, scanning electron microscopy was performed on cells treated with either MN58 or RSM-932A (Fig. 5). MN58 was found to modulate the shape of the cell wall giving the wall a bumpy surface (Fig. 5B) while RSM-932A did not greatly affect the normally smooth surface of the cell (Fig. 5C). Taken together with the data from the autolysis experiments, it seems that smaller assembly sizes of LTA and/or increased LTA production leads to distortion of the cell wall and sensitivity to autolysis. Meanwhile, increasing the quantity of larger assembly sizes of LTA does not lead to cell wall distortion and does not affect the level of sensitivity of the cell to autolytic induction.

### In enzymatic assays with *S. pneumoniae* extracts MN58b is still an inhibitor but RSM-932A becomes an agonist

**Figure 6:**
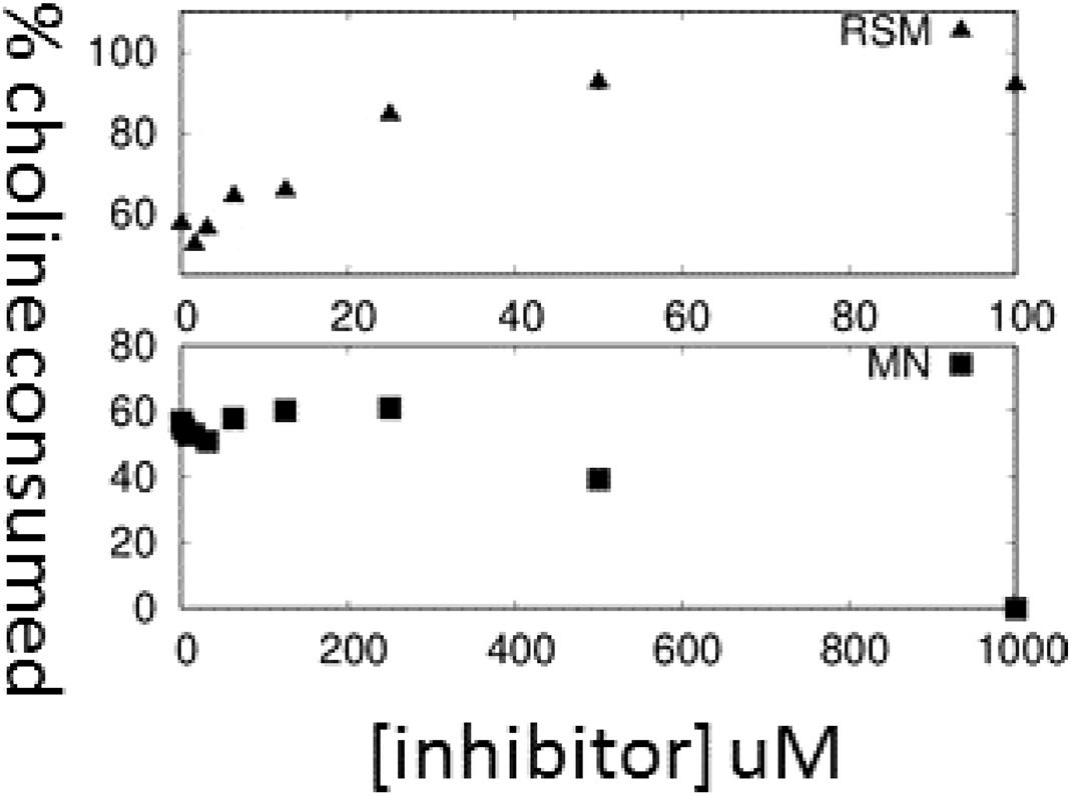
Choline consumption in the presence of RSM-932A (top) and MN58b (bottom).

In light of the inconsistent results between sChoK inhibition by MN58b and RSM-932A which are both known choline kinase inhibitors, we repeated the colorimetric experiments using *S. pneumoniae* extracts as the source of sChoK, The goal of the experiment was to determine whether or not differences in sChoK inhibition could be detected in a more native environment than that provided by extracts of *E. coli* overexpressing sChoK or purified sChoK. MN58b continued to function as an inhibitor of choline kinase activity under these conditions (Fig. 6, bottom) Surprisingly we found that, while RSM-932A was somewhat inhibitory at lower concentrations, it functioned as an agonist of choline kinase activity at higher concentrations in a dose dependent manner (Fig. 6, top). This agonism was confirmed by mass spectrometry (data not shown) and was also found to be ATP dependent, thereby supporting the inference that RSM-932A was still affecting sChoK activity, although in the opposite direction. This suggested that there was a factor found in the *S. pneumoniae* cells that was not found in *E. coli* cells nor in the purified enzyme that modified how sChoK interacted with RSM-932A.

## Discussion

We demonstrated in this study that purified sChoK could potentially be used as a screening system to detect inhibitors of *S. pneumoniae* growth, and we presented evidence of the strongest sChoK inhibitors of enzymatic action and cell growth described to date. In the case of MN58b, the MIC is at least a magnitude of order smaller than its IC_50_, which suggests that either the effect of this drug is occurring on other proteins or that the effect of the drug increases as a consequence of being accumulated at a high concentration inside a cell. In the case of RSM-932A, both the IC_50_ and MIC are in the sub-micromolar range, which would normally suggest that the inhibitory effect of the drug was due to its binding to its enzyme target alone. However, in a more native environment, RSM-932A actually functions as an agonist.

Importantly, we did not observe the suppression of downstream lipoteichoic acid production that is the expected consequence of choline kinase inhibition^17^ due to the putative attenuation of phosphocholine production which would remove one of the building blocks of LTA Instead, we observed increased phosphocholine production (in the case of MN58b) and changes in lipoteichic acid sizes to either smaller (MN58b) or larger sizes (RSM-932A). This change appears to have an effect on cell sensitivity to autolysis and on the shape of the cell wall. Dysregulation of LTA should lead to differences in sensitivity because prior to cleaving peptidoglycan, autolysins must first bind the phosphocholine head of LTA. However, the question remains as to whether changes in LTA assembly is a consequence of inhibitor action on choline kinase or rather a separate process, such as direct binding of RSM-932A and MN58b to a yet unidentified protein responsible for polymerization of LTA chains or even to a separate choline binding protein entirely.

Interestingly, there is a key difference in autolysis, which may occur because of overexpression of LTA and/or overexpression of smaller LTA sizes, leading to differences in cell wall shape as well. Larger sizes of LTA do not appear to lead to changes in sensitivity to autolysis nor to changes in the cell wall. However, in both cases, changes in the size of LTA are associated with the effectiveness of the drugs in inhibiting cell growth.

We would thus like to propose a possible model that could both explain the disparate results with RSM-932A in its activity as either an inhibitor or an agonist and the differences in LTA polymerization and production observed between MN58b and RSM-932A: choline kinase forms part of a larger complex that includes the protein responsible for LTA chain assembly. In fact, hChoKα has also been proposed to be a scaffolding protein in eukaryotic cells^20^. A similar function could be anticipated for sChoK.

The differences in RSM-932A action cannot be explained by positing that this action differs depending on whether or not sChoK is in the presence of a cell extract because, in the presence of an E. coli cell extract, this drug remains an inhibitor. In the presence of an *S. pneumoniae* extract, however, this drug becomes an agonist. One possibility is that sChoK, which localizes to the cytoplasm^21^, forms a complex with another cellular element that is not found in *E. coli*, and this interaction is modulating the effects of RSM-932A. We thus propose that this other cellular element is an LTA polymerase (which may or may not localize to the cytoplasm) and that this interaction is a two-way process. Inhibitor binding to sChoK may also modulate this LTA polymerase indirectly via this putative protein–protein interaction. Meanwhile, MN58b and RSM-932A, while both inhibitors of sChoK, do not have the same mechanism of action. As seen from the steady-state experiments, these two inhibitors bind to sChoK differently. MN58b is antagonistic to both ATP and choline, while RSM-932A is antagonistic only to choline. A different mode of binding would have a different effect on the structure of sChoK and therefore lead to disparate effects of these drugs on the putative interaction with a putative LTA polymerase, which would, in turn, lead to outputs in LTA molecule sizes that differ. In fact, MN58b and RSM-932A are found to have a differential mode of interaction with hChoKα as well^14,22^.

It remains to be seen whether this model can be supported through proteomic studies that could identify the putative sChoK complex or whether or not these experiments could lead to identification of the enzyme responsible for LTA polymerization and the mechanism whereby dysregulating production and polymerization of LTA leads to inhibition of cell division.

However, the mechanism of action of any choline analog could include simultaneously (or exclusively) inhibiting the action of bacterial choline binding proteins (CBP) other than choline kinase. These include lysozyme (LytC), Amidase (CbpD), choline binding protein (CbpF), pneumococcal surface protein A and C (PspA, PspC), choline transporters, and Phosphorylcholine esterase (Pce). LytC, CbpD, and CbpF are all involved in the autolytic process. Our study has shown that MN58b and RSM932A neither enhance, nor block the process of autolysis, making the possibility that LytC, CbpD and CbpD are direct targets unlikely. PspA, PspC, and Pce are known to manage cell virulence, but it is unclear if these are essential proteins and none have been validated as drug targets for blocking cell growth. Nevertheless it is certainly possible that there is either MN58b and RSM932A directly targets some choline binding protein or even modulates expression of these proteins to the point where growth is disabled^23^. Though we continue to favor the model that choline kinase is a target of choline kinase inhibitors, to truly begin distinguishing between the different possible targets, a good first step would be to identify mutations in strains resistant to MN58b and RSM932A and/or monitor changes in expression that are a result of treatment. Nevertheless, it is clear that by using recombinant purified or unpurified sChoK as a system of screening, one can identify promising new inhibitors of *S. pneumoniae* growth to be developed as possible antibiotics.

A valid concern of applying sChoK inhibitors to treat an infection is that hChoK activity could be affected, leading to secondary effects. This concern is particularly valid for those inhibitors that have been developed to block hChoK. As a starting point, we deliberately chose the best characterized two hChoK inhibitors available for study, MN58b and RSM-932A. This includes pre-clinical toxicity studies, that give us an idea of dosage limits^24^. The effectiveness of these drugs against infection in animal and human models within the limits of toxicity remains to be assessed. We do know that these drugs are non-toxic to human primary cell lines and selective to tumor cells. RSM-932A even reached phase I drug trials for treating tumors (https://clinicaltrials.gov/ct2/show/NCT01215864). In addition, these drugs are being examined for use inflammatory disorders^25,26^. What we have determined here is that that the usefulness of sChoK inhibitors, including those designed for hChoK, could extend to use as antibiotics for treating *S*. *pneumoniae* infections. In addition, possible toxicity could be prevented in future studies by designing sChoK inhibitors that are selective for bacterial isoforms.

## Supplementary information

Supplementary Figure.
