# Supplementary figures and images for: Identification and validation of novel and more effective choline kinase inhibitors against Streptococcus pneumoniae
Source: Sci Rep. 2020 Sep 22;10:15418. doi: 10.1038/s41598-020-72165-6 (PMC7508948; doi:10.1038/s41598-020-72165-6)

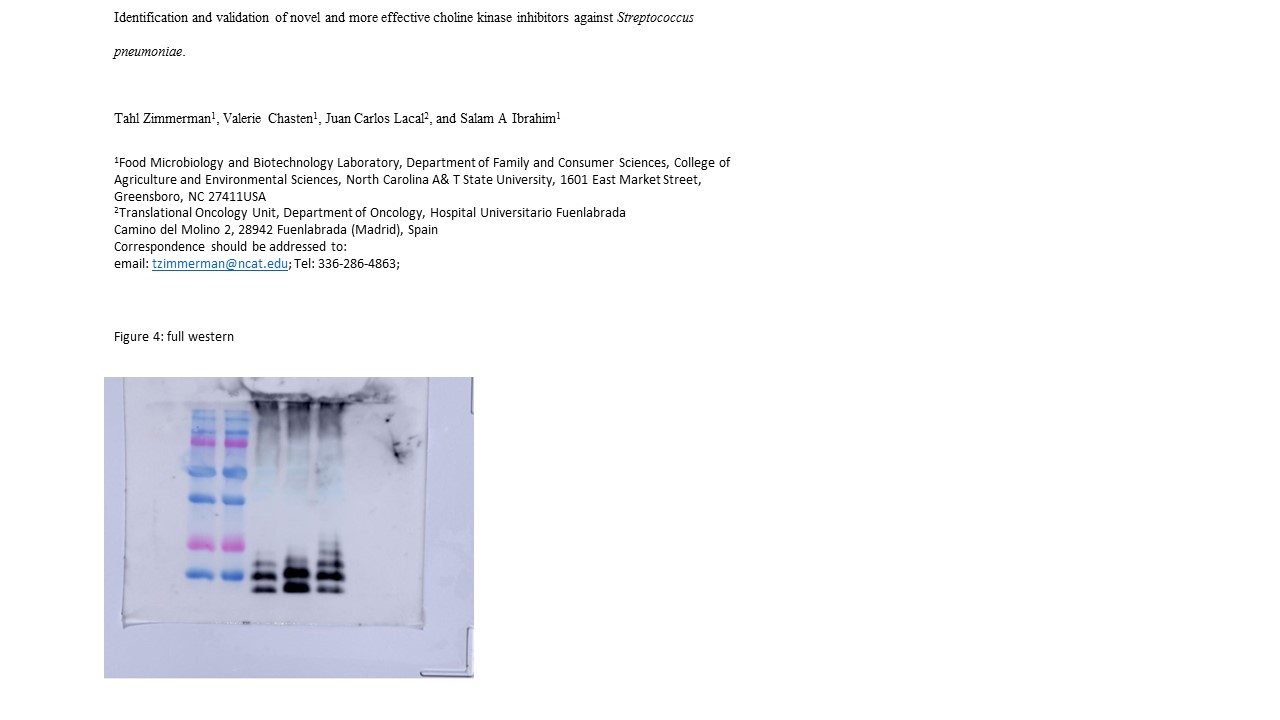

Supplement: Supplementary file 1 — Supplementary Figure. [file 41598_2020_72165_MOESM1_ESM.jpg]
